# Supplementary material for: Insight into the Reaction of Alexidine with Sodium Hypochlorite: A Potential Error in Endodontic Treatment
Source: Molecules. 2021 Mar 15;26(6):1623. doi: 10.3390/molecules26061623 (PMC7999803; doi:10.3390/molecules26061623)
Supplement: Supplementary file 1 [file molecules-26-01623-s001.pdf]

Supplementary material

The insight into the reaction of alexidine with sodium hypochlorite – a potential error in endodontic treatment

Barbara Czopik [1]*, Monika Ciechomska[2], Joanna Zarzecka[1], Maciej Góra[3] and Michał Woźniakiewicz[2]

[1] Department of Operative Dentistry with Endodontics, Institute of Stomatology, Jagiellonian University Medical College in Kraków, Montelupich 4, 30-155 Kraków, Poland; e-mail: bczopik@o2.pl, j.zarzecka@uj.edu.pl

[2] Laboratory for Forensic Chemistry, Department of Analytical Chemistry, Faculty of Chemistry, Jagiellonian University, Gronostajowa 2, 30-387 Kraków, Poland; e-mail: monikaciechomska@wp.pl, michal.wozniakiewicz@uj.edu.pl

[3] Department of Organic Chemistry, Faculty of Chemistry, Jagiellonian University, Gronostajowa 2, 30-387 Kraków, Poland; e-mail: maciej.gora74@gmail.com

* Correspondence: bczopik@o2.pl ; Tel.: +48 608505816

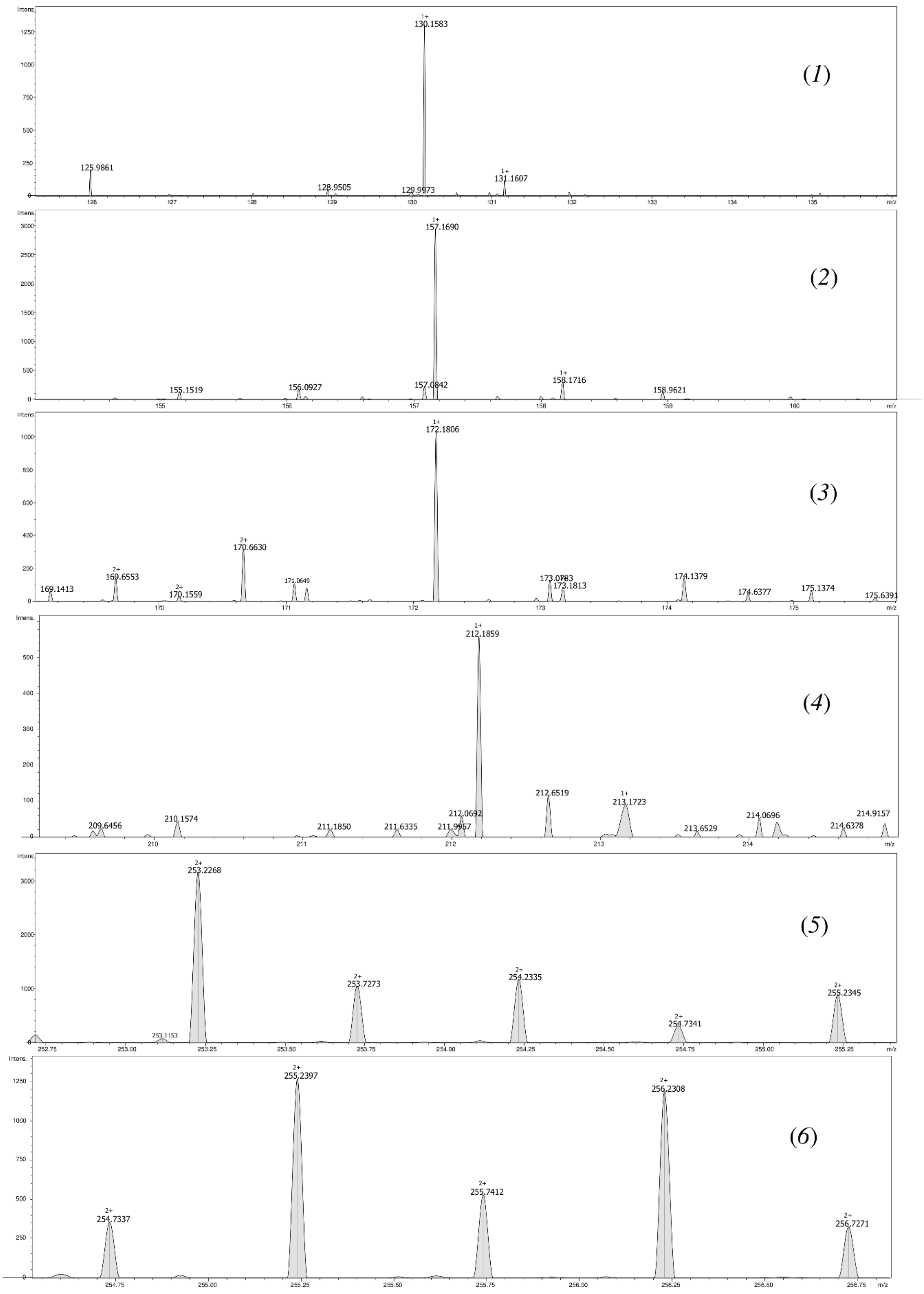

Fig. 1-S. Mass spectra of investigated compound generated during the reaction between ALX and NaOCl. See Table 1 in the main manuscript for compounds 1-6.

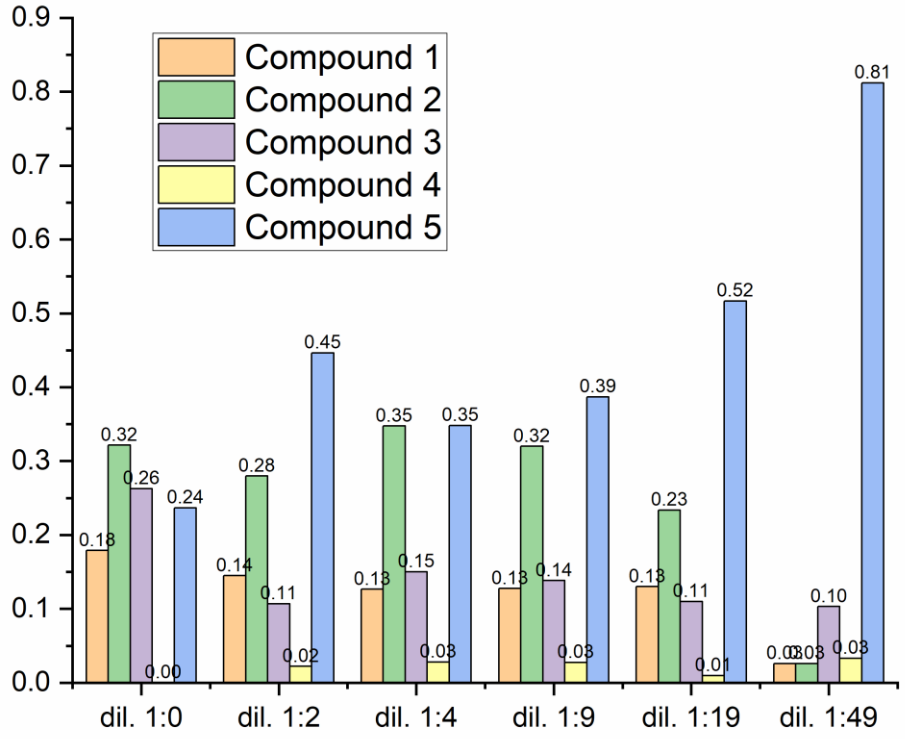

Fig. 2-S Relative abundance of investigated ALX – NaOCl reaction products depending on dilution of 5.25% (w/v) NaOCl reagent (in v/v).
